# Green Extraction of Lotus Leaf (*Nelumbo nucifera Gaertn*) Polyphenols: Unraveling the Mechanism of Ultrasound-Assisted Deep Eutectic Solvents

**DOI:** 10.3390/foods14234045

**Published:** 2025-11-25

**Authors:** Jing Sun, Mengqi Qin, Luyang Chen, Xin Li, Xinyan Wu, Gang Ye, Jianjun Deng, Haixia Yang

**Affiliations:** 1 College of Food Science and Nutritional Engineering, China Agricultural University, Beijing 100083, China; 2 College of Veterinary Medicine, Sichuan Agricultural University, Chengdu 611130, China; 3 State Key Laboratory of Vegetable Biobreeding, Institute of Vegetables and Flowers, Chinese Academy of Agricultural Sciences, Beijing 100081, China; 4 Shaanxi Key Laboratory of Degradable Biomedical Materials, Shaanxi R&D Center of Biomaterials and Fermentation Engineering, Biotech & Biomed Research Institute, School of Chemical Engineering, Northwest University, Xi’an 710069, China

**Keywords:** deep eutectic solvents, total phenolic content, extraction mechanism, antioxidant activity

## Abstract

Deep eutectic solvents (DESs) have attracted considerable attention in recent years because of their cost-effectiveness, safety, and sustainability. In this study, we developed 19 DESs for the extraction of antioxidant polyphenolic compounds from lotus leaves, utilizing ultrasound-assisted extraction (UAE). Among the DESs examined, choline chloride (ChCl) and lactic acid (ChCl: lactic acid) exhibited the highest extraction efficiency. The optimal conditions were established as follows: molar ratio of 1:2.6, solid-to-liquid ratio of 1:20 g/mL, water content of 8%, and ultrasound time of 65 min, which proved to be more efficient than conventional extraction methods such as water and ethanol. Under the optimal conditions, the total phenolic content (TPC) was 187.23 ± 14.67 mg GAE/g DW, and the extracts exhibited high antioxidant activity (DPPH IC_50_: 0.92 ± 0.23 mg/mL; FRAP: 21.56 ± 3.05 mg Trolox/g DW). This superiority arises from the formation of robust hydrogen bonds between ChCl and lactic acid, in conjunction with improved mass transfer efficiency. This study provides a green alternative method for polyphenol extraction from lotus leaves.

## 1. Introduction

*Nelumbo nucifera Gaertn*. (lotus), a member of the Nymphaeaceae family, is widely distributed across Eastern Asia, China, and India, where the total area devoted to its cultivation is estimated to exceed 3344 km^2^ [1]. Lotus is often undervalued for its medicinal properties [2], as most individuals regard it merely as an economic ornamental, which leads to a considerable waste of potential resources. Importantly, >80 flavonoids and >10 phenolic acids have been identified in lotus leaves, with 2.9% of flavonoids (fresh weight) and 3.5% of phenolic acids (dry fractions) in whole leaves [3,4,5]. As a rich source of polyphenols with antioxidant properties, the extracts of lotus leaves offer great potential for resource recycling and functional food development. However, despite an abundant annual yield of 70–100 M tons [6], lotus leaf remains largely underutilized, with only 1% been used in the food industry. To produce extracts suitable for such applications, there is a pressing need to establish suitable extraction methods based on the matrix source and properties of polyphenolic compounds. The choice of solvent significantly influences extraction efficiency and compound activity.

Traditional extraction methods, including Soxhlet and solid–liquid extraction, are frequently supplanted by alternative techniques due to several limitations: (1) they may require up to 24 h (or longer), (2) they limit the quantity of samples processed simultaneously, and (3) they necessitate the use of organic solvents such as methanol, ethanol, and diethyl ether [7,8,9,10].

To overcome these limitations, advanced extraction methods have been developed, including supercritical fluid, microwave-assisted (MAE), and ultrasound-assisted extraction (UAE). These techniques primarily improve extraction efficiency by minimizing solvent volume and reducing extraction time [11,12]. Given considerations of cost and equipment availability, MAE and UAE are more commonly utilized [13] by disrupting plant cell walls through cavitation or thermal shock, these methods enhance yields; for instance, flavonoid yields are up to 20–30% greater than traditional solvent extraction [14]. However, solvent selection is a key factor affecting the extraction capacity of UAE.

Notably, deep eutectic solvents (DESs), formed by hydrogen bond acceptors (HBAs) and hydrogen bond donors (HBDs), are frequently employed to extract bioactive compounds from botanical sources owing to their biodegradability and low toxicity [15]. The DES systems display similar properties to ionic liquids. However, DES synthesis does not rely on chemical reactions but rather on the formation and disruption of hydrogen bond networks [16,17]. Due to most DESs exhibiting high viscosity, the addition of moderate amounts of water can improve extraction efficiency by lowering viscosity [18].

Due to comparable polarities and shared chemical interactions, including hydrogen bonding, dipole–dipole forces, and van der Waals forces, DESs have been used in efficient polyphenol extraction from plants, such as fruit or leaf waste from *Capparis ovata*, *Moringa oleifera* L., Mulberry, *Pollen Typhae*, *Lycium barbarum* L., and lemon verbena [19,20,21,22,23,24]. Although DESs have been utilized to extract polyphenolic compounds from lotus leaves, achieving a yield of 126.10 mg/g, the HBD: propylene glycol employed has been deemed unsuitable for polyphenol extraction due to the steric hindrance it introduces [25]. The steric hindrance resulting from the conformation of DESs suggests elevated surface tension, necessitating a higher energy input for the active compound to interact with the HBA. To overcome this limitation, the present study evaluated the ultrasound-assisted extraction of polyphenols from lotus leaves utilizing 19 different DES systems. DES systems based on choline chloride (ChCl), betaine, and L-proline were compared for their efficacy in extracting polyphenols from lotus leaves.

Experimental variables can significantly influence the extraction efficiency of DESs, necessitating a robust experimental design to evaluate the impact of each variable and optimize conditions for maximizing the yield of extracted polyphenols. Accordingly, the Box–Behnken design was employed to assess the effects of various variables on the final polyphenol yield and bioactive properties. This study also aims to further elucidate the potential mechanism underlying polyphenol extraction using the UAE and DES system. This research constitutes the first investigation into the mechanism of combined UAE and DES extraction of polyphenols from lotus leaves.

## 2. Materials and Methods

### 2.1. Materials and Chemicals

**Figure 1:**
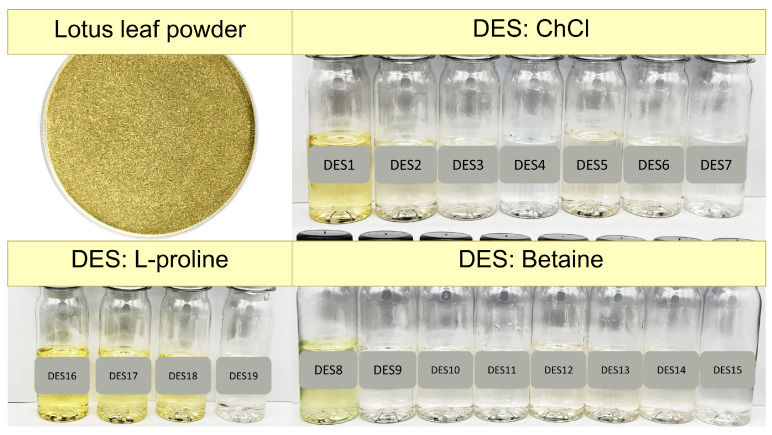
Images of dried lotus leaf powder and the prepared DES systems composed of choline chloride (ChCl), betaine, and L-proline as HBAs.

Lotus leaves sourced from Anhui Herbal Medicine Cultivation Co., Ltd. (Haozhou, China) were dried at 60 °C for 24 h in an oven (GZX-9030MBE, Boxun Medical Biological Instrument Corp., Shanghai, China), crumbled, sieved (using a 40-mesh sieve), and then stored at 4 °C for further analysis (Figure 1).

Choline chloride (ChCl, 98%), betaine (98%), L-proline (98%), levulinic acid (95%), lactic acid (95%), urea (98%), glycerol (98%), D-glucose (≥98%), maltose (≥98%), sodium acetate (≥99%), and ferric chloride (≥99%) were purchased from Aladdin Scientific Co., Ltd. (Shanghai, China). 2,2-Diphenyl-1-(2,4,6-trinitrophenyl) hydrazyl (DPPH), 2,4,6-tri(2-pyridyl)-s-triazine (TPTZ), (±)-6-hydroxy-2,5,7,8-tetramethylchromane-2-carboxylic acid (Trolox), gallic acid (99.9%), and Folin–phenol reagent were obtained from Shanghai Yuanye Biotechnology Co., Ltd. (Shanghai, China).

### 2.2. Preparation of Deep Eutectic Solvents

The DES-1 to DES-7, DES-8 to DES-15, and DES-16 to DES-19 (Figure 1) were synthesized using choline chloride, betaine, and L-proline as the hydrogen bond acceptors (HBAs) in combination with various hydrogen bond donor (HBD) compounds such as levulinic acid, lactic acid, urea, glycerol, D-glucose, and maltose. The molar ratio of HBAs/HBDs was recommended according to the literature (as shown in Table 1). To ensure complete dissolution, 2 mL of distilled water was uniformly added to all systems and then they were heated at 80 °C for 1–40 min with constant agitation at 500 rpm until a homogeneous liquid was achieved.

### 2.3. Determination of Physical Properties of Deep Eutectic Solvents

The pH was determined using a pH meter (FE28-Micro, METTLER TOLEDO, Greifensee, Switzerland). Density was calculated using the following formula:

(1) $$\rho_{(DES)}=\frac{m_{3}-m_{1}}{m_{2}{\;-\;m}_{1}}\times \rho_{(H_{2}O)}$$

where m_1_, m_2_, and m_3_ are the weight of the bottle, the bottle with water, and the bottle with DES, respectively.

Rheological analysis was conducted utilizing a rheometer (DHR-2, TA Instruments, New Castle, DE, USA) equipped with parallel plate geometry (25 mm diameter) and a 1 mm gap between plates. The samples underwent deformation speeds ranging from 0.1 to 100 s^−1^ at 25 °C. The reported value is the mean of five measurements.

### 2.4. Determination of Total Phenolic Content and Antioxidant Capacity

The total phenolic content (TPC) was determined by the Folin–Ciocalteu colorimetric method reported by Elaine Benítez-Correa [26] with some modifications. A 0.1 mL aliquot of sample was diluted in 4.9 mL of distilled water, then mixed with 1 mL of Folin–Ciocalteu reagent and allowed to stand for 3 min. Subsequently, 4 mL of a 0.94 M aqueous sodium carbonate solution was added, the mixture was shaken vigorously, and it was incubated in the dark at 50 °C for 5 min. Following centrifugation at 4000× rpm for 1 min, the absorbance of supernatant was measured at 765 nm using a spectrophotometer (UV–Visible V-5600, Yuanxi Instruments Co., Ltd., Shanghai, China). Calibration curves were established with gallic acid standards (y = 6.64686x + 0.00586; R^2^ = 0.9987), and the results were expressed as mg gallic acid equivalent per gram of dry weight (mg GAE/g DW).

The 2,2-diphenyl-1-(2,4,6-trinitrophenyl) hydrazyl (DPPH) free radical scavenging method was determined according to the detailed assay described by Hee Nam [27] with some modifications. The five extracts (water, 75% EtOH, DES-2, DES-15, and DES-19) were prepared at a concentration of 50 mg/mL and diluted in water to final concentrations of 2.5, 1.67, 1.25, 1, 0.83, and 0.71 mg/mL. A total of 100 µL of each sample was mixed with 2.9 mL of DPPH solution (0.025 mg/mL, dissolved in methanol). The mixture was reacted in the dark at room temperature for 30 min. Distilled water served as the negative control and the absorbance was measured at 517 nm with a spectrophotometer (UV–Visible V-5600, Yuanxi Instruments Ltd., Shanghai, China). All determinations were performed in triplicate. The percentage inhibition of DPPH radical by extracts was based on the following:

(2) $$\mathrm{Inhibition}\;(\%)=\frac{A_{0}\;-\;A_{t}}{A_{0}}\times 100$$

where A_0_ is the control and A_t_ is the absorbance of sample. The IC_50_ values (mg/mL, the concentration of the test samples required to reach inhibition of DPPH radical to 50%) were calculated.

Ferric-reducing antioxidant power (FRAP) was determined following the protocol outlined by Noor Alsaud [28] with slight modifications. Specifically, 400 µL of the sample was combined with 3.6 mL of TPTZ working solution, which was prepared by mixing sodium acetate buffer (300 mM pH 3.6), TPTZ (10 mM) and FeCl_3_·6H_2_O (20 mM) in a ratio of 10:1:1. The mixture was incubated in the dark for 30 min. Absorbance was measured at 593 nm using a spectrophotometer (UV–Visible V-5600, Yuanxi Instruments Ltd., Shanghai, China). Calibration curves were established with Trolox standards (y = 6.432x − 4.012, R^2^ = 0.9981), and the results were expressed as mg Trolox equivalent per gram of dry weight (mg Trolox/g DW).

### 2.5. Extraction Procedure

The extraction procedure was conducted following previously reported methods with some modifications [26]. A sample (0.50 g) was combined with 10 mL of DES, then sonicated at 30 °C for 30 min (40 kHz, 120 W) (JP-040S ultrasound, Skymen, Shenzhen, China). To prevent an increase in temperature during ultrasonication, the water in the ultrasonic bath should be drained and replaced with room-temperature distilled water every 5 min. The obtained mixture was centrifuged at 10,000× rpm for 10 min, and the supernatant was collected for further analysis.

### 2.6. Optimization of Extraction Process

Single-factor design experiments were conducted under various conditions: (a) molar ratios of HBA/HBD (1:1, 1:2, 1:3, 1:4, 1:5); (b) water content (0%, 10%, 20%, 30%, and 50%); (c) solid-to-liquid ratios (1:10, 1:15, 1:20, 1:25, and 1:30 g/mL); and (d) ultrasonic time (30, 40, 50, 60, and 70 min) (40 kHz,120 W) to assess the yield of TPC.

Based on single-factor experiments, a four-factor, three-level response surface methodology (RSM) was employed to optimize the extraction of total polyphenols from lotus leaves. The factors investigated included the HBD: HBA molar ratio (1, 2, 3), liquid–solid ratio (15, 20, 25 mL/g), ultrasonic time (50, 60, 70 min), and water content (0, 10, 20%). The TPC (mg GAE/g DW) served as the response variable. Data analysis was conducted using Design Expert 13.0. The experimental results are presented in Table 2, accompanied by the following quadratic regression equation:

Yield of TPC = 185.29 + 28.74A + 13.14C − 15.33AC − 31.71AD − 16.17BC − 22.70A^2^ − 14.24B^2^ − 15.22C^2^ − 39.07D^2^

where A represents the molar ratio of HBA/HBD, B represents the liquid–solid ratio, C represents the ultrasonic time, and D represents the water contents.

### 2.7. Fourier Transform Infrared Spectrometer

The functional groups of ChCl, DL-lactic acid, and ChCl–lactic acid (molar ratio 1:2) were analyzed using a Shimadzu IRTracer-100 FTIR spectrometer (IRTracer100, Shimadzu Co., Ltd., Kyoto, Japan), which is equipped with an MIR-TGS detector. Spectra were recorded at room temperature over the range of 4000–500 cm^−1^, with 16 scans accumulated at a resolution of 4 cm^−1^.

### 2.8. Scanning Electron Microscope

Scanning electron microscope (SEM) analysis was performed using a Hitachi SU5000 (Hitachi SU5000, Hitachi High-Tech Co., Ltd., Shanghai, China), equipped with a secondary electron detector and operated at an acceleration voltage of 20 kV. The samples analyzed comprised raw lotus leaf powder and materials extracted using DES and ethanol (75%) under optimal extraction conditions determined by RSM analysis.

### 2.9. Statistical Analysis

All measurements were conducted in triplicate and are presented as mean ± SD. One-way analysis of variance (ANOVA) was performed using Graphpad Prism 9.0 (San Diego, CA, USA). The BBD-RSM design was executed using Design-Expert 13.0 (Minneapolis, Minnesota, MN, USA). ANOVA was applied to assess linear regression, quadratic coefficients, and interactions. The predicted R^2^ and adjusted R^2^ were estimated at the 95% significance level based on the polynomial equation (*p* < 0.05).

## 3. Results and Discussion

### 3.1. Characterization of Different DESs

Table 1 summarizes the DES systems commonly used in recent years for polyphenol extraction. Choline chloride, betaine, and L-proline were identified as the most frequently employed HBAs, and were, therefore, selected as the HBAs in this study. HBDs, including carbohydrates, alcohols, acids, and amides, were chosen based on their prevalence in reported applications. We further characterized the physicochemical properties (e.g., pH, viscosity, and density) of the resulting DES combinations. This characterization was conducted to establish a correlation between DES properties and polyphenol extraction efficiency, thereby providing a scientific basis for the optimization of DES-based extraction.

As illustrated in Figure 1, the 19 DESs generally display fluidity and notable variations in visual appearance at room temperature. Their properties, including pH, density, and viscosity, are summarized in Table 3. Viscosity, which directly influences a liquid’s flow resistance, is typically elevated in DESs due to complex hydrogen bonding between the HBAs and HBDs [29]. This high viscosity poses challenges for industrial applications. Enhanced hydrogen bonding between HBAs and HBDs results in increased viscosity, which restricts molecular mobility [30]. DESs with higher viscosities (>200 mPa·s) demonstrate greater ionic hydrophobicity [31], including ChCl-based DES-1, DES-3, DES-5, and DES-6, as well as betaine-based DES-8 and DES-9, and L-proline-based DES-17. In contrast, DESs with lower viscosities (41.3–167.4 mPa·s; DES-2, DES-10, DES-14, DES-16) may exhibit reduced surface tension, thereby facilitating polyphenol extraction. However, excessively low viscosities (4.8–20 mPa·s; DES-4, DES-7, DES-15, DES-19) suggest weak intermolecular interactions, which also inhibit extraction.

Density is a fundamental physical property of DESs. Solvents with densities of ≤1.0 g/cm^3^ are generally preferred for extraction processes due to their compatibility with water [31]. As indicated in Table 3, DES-1, DES-2, DES-12, and DES-15 meet this criterion (≤1.14 g/cm^3^), thereby satisfying most extraction requirements. However, polyhydroxylated HBDs, such as glycerol, D-glucose, and maltose, increase the density of DESs [32]. Consequently, formulations containing these HBDs, including DES-3, DES-4, DES-5, DES-6, DES-10, DES-11, DES-13, DES-14, DES-16, DES-17, and DES-18, exhibit elevated densities ranging from 1.15 to 1.29 g/cm^3^.

The pH value of DES significantly affects the efficiency of polyphenol extraction, which is determined by the acidity and basicity of HBA and HBD [33]. Bubalo et al. [34] demonstrated that acid-based DESs achieve higher recovery of free anthocyanins from grape skins compared to sugar-based alternatives. The acidic conditions can improve the stability of polyphenols and increase their extraction rates. Consequently, acidic HBDs such as levulinic acid (DES-1, DES-8) and lactic acid (DES-2, DES-9) are likely to enhance polyphenol extraction in this study. Given the properties of DESs, including low viscosity, water-like density, and acidic pH, DES-2 (ChCl–lactic acid) appears to be particularly suitable for the extraction of polyphenols from lotus leaves. Although these physicochemical trends are apparent, objective empirical validation would further strengthen the evidence.

### 3.2. Determination of the Optimal DES for Polyphenol Extraction

#### 3.2.1. Screening of DESs for Enhanced Polyphenol Extraction

**Figure 2:**
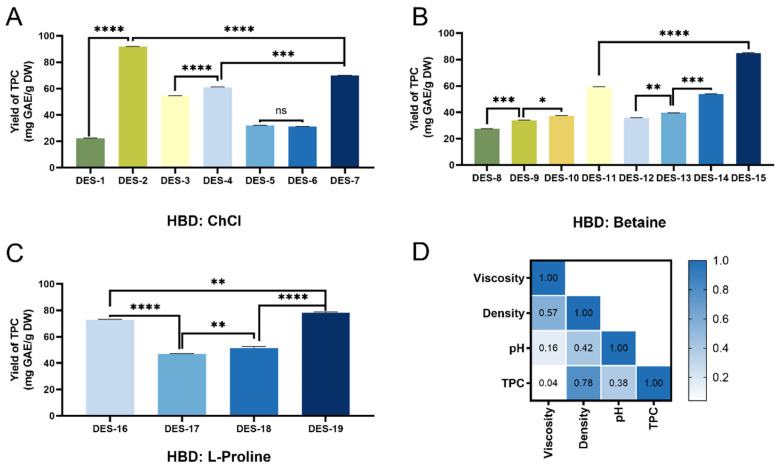
Total phenolic content (TPC) extracted from different DES combinations based on (**A**) ChCl, (**B**) betaine, and (**C**) L-proline. “ns” indicate not significant, “*”, “**”, “***”, and “****” indicate significant differences (*p* < 0.05, <0.01, <0.001, and <0.0001, respectively). (**D**) Pearson correlation analysis between TPC and viscosity, density, and pH. Values represent correlation coefficients.

Figure 2A–C reveals significant differences in polyphenol content extracted by ChCl-, betaine-, and L-proline-based DES systems. Among these, DES-2 (ChCl–lactic acid), DES-15 (betaine–urea), and DES-19 (L-proline–urea) achieved high extraction efficiencies, yielding 84.52 ± 0.21, 77.60 ± 0.49, and 71.12 ± 0.47 mg GAE/g DW, respectively. DES-2 (ChCl-lactic acid) demonstrated the highest polyphenol content, consistent with the conclusions drawn in Section 3.1. These findings align with the results reported by Saha et al. [35]. L-proline-based DESs generally outperformed betaine-based DESs, which can be attributed to the abundant hydrogen bond acceptors in L-proline that facilitate bond formation. Meanwhile, ChCl-, betaine-, and L-proline–glycerol DESs exhibited comparable TPC values, indicating similar polyol-mediated behavior. It is worth noting that selecting DES-2, DES-15, or DES-19 for further research requires additional evaluation of their antioxidant activity levels.

Figure 2D illustrates the Pearson correlation coefficients between the physicochemical properties of DES and TPC, where the values presented are correlation coefficients. Viscosity exhibits a strong negative correlation with TPC (r = 0.86, *p* < 0.05). Notably, D-glucose and glycerol produced medium-to-high viscosities, which can be attributed to the hydrogen bonding between HBD and HBA that restricts molecular mobility [31]. The system pH may also influence extraction efficiency, and acidic conditions generally favor polyphenol extraction.

#### 3.2.2. Comparison of the Effects of Different Extraction Methods on Antioxidant Activity

**Figure 3:**
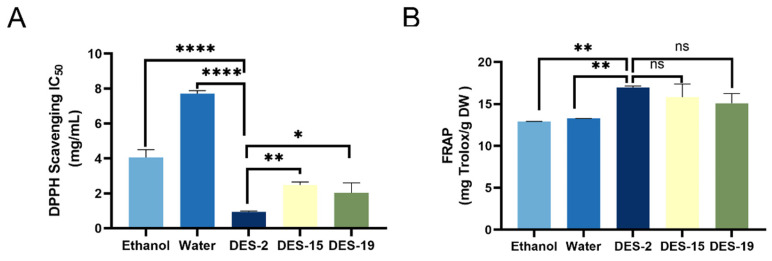
(**A**) DPPH radical scavenging capacity (IC_50_) and (**B**) ferric reducing antioxidant power (FRAP) of extracts obtained using water, 75% ethanol, DES-2, DES-15, and DES-19. “ns” indicate not significant, “*”, “**”, and “****” indicate significant differences (*p* < 0.05, <0.01, and <0.0001, respectively).

The antioxidant activities of five extracts, derived from water, 75% ethanol, DES-2 (ChCl: lactic acid), DES-15 (betaine: urea), and DES-19 (L-proline: urea), were evaluated through DPPH radical scavenging and FRAP total reduction capacity assays. As illustrated in Figure 3, the extract obtained using DES-2 (ChCl: lactic acid) exhibited an IC_50_ value of 0.96 ± 0.04 mg/mL, significantly lower than those of the extracts from 75% ethanol (4.06 ± 0.45 mg/mL) and water (7.72 ± 0.16 mg/mL) (*p* < 0.0001). Additionally, the IC50 value for the DES-2 (ChCl: lactic acid) extract was also significantly lower than those for DES-15 (betaine: urea, 2.46 ± 0.17 mg/mL) and DES-19 (L-proline: urea, 2.03 ± 0.57 mg/mL) (*p* < 0.05), indicating that the extract from DES-2 (ChCl: lactic acid) exhibited the highest DPPH radical scavenging capacity. Moreover, the FRAP values further confirmed that the extract from DES-2 (ChCl: lactic acid, 16.94 ± 0.20 mg Trolox/g DW) demonstrated the strongest reducing capacity among the tested solvents.

Soukaina et al. [36] demonstrated that ChCl-based DESs exhibit significantly higher efficiency in extracting polyphenols compared to traditional solvents such as methanol. This finding suggests that the polarity of ChCl-based DESs is closely associated with the polarity of polyphenolic compounds in plants, thereby directly affecting their solubility. Furthermore, the carboxyl group in lactic acid can form hydrogen bonds with polyphenolic substances [37,38,39]. Reports indicate that the ChCl: lactic acid combination serves as the most effective solvent for recovering phenolic compounds Consequently, DES-2: ChCl: lactic acid will be selected for subsequent studies in this research.

### 3.3. Optimization of DES Extraction Conditions

#### 3.3.1. Single-Factor Test

The extraction efficiency of the UAE-DES system is influenced by several factors, including molar ratio, liquid–solid ratio, ultrasound time, and water content. To evaluate the effect of the HBA/HBD molar ratio on TPC, experiments were conducted across a range from 1:1 to 1:5 while keeping other conditions constant (solid–liquid ratio of 1:20 g/mL, ultrasound duration of 30 min, and water content of 20%). This range was chosen in accordance with previous research [25], which suggests that an imbalance in HBDs or HBAs may influence phenolic compound yield. TPC rose markedly as the molar ratio of ChCl to lactic acid increased from 1:1 (79.36 ± 2.37 mg GAE/g DW) to 1:2 (147.30 ± 6.22 mg GAE/g DW), but subsequently declined from 1:2 to 1:5 (102.10 ± 1.75 mg GAE/g DW). This trend may be attributed to excessive acidity and elevated viscosity resulting from a surplus of lactic acid, which could promote polyphenol degradation and impede mass transfer efficiency [40]. Based on these results, a ChCl–lactic acid molar ratio of 1:2 was chosen for further experiments.

**Figure 4:**
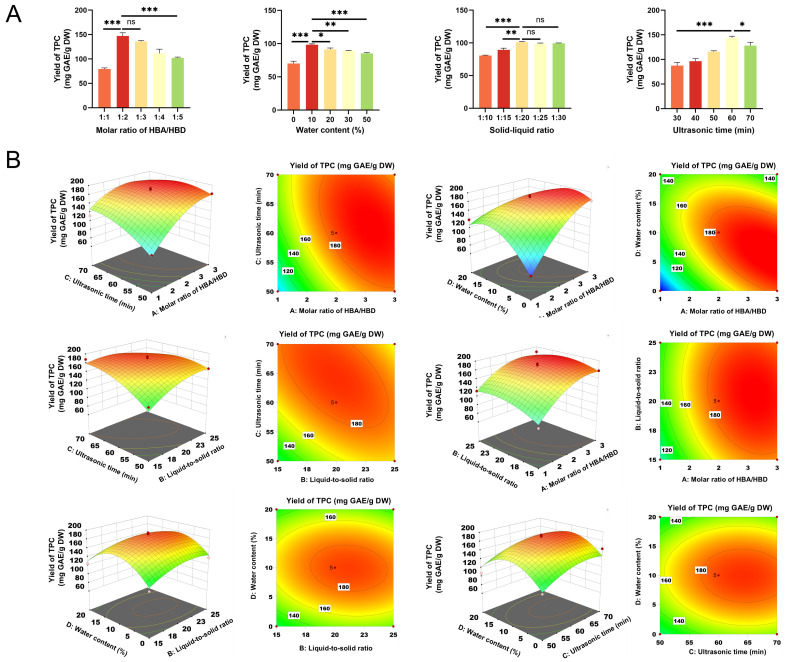
(**A**) Effects of molar ratio, water content, solid-to-liquid ratio, and ultrasonic time on TPC yield in single-factor experiments. “ns” indicate not significant, “*”, “**”, and “***” indicate significant differences (*p* < 0.05, <0.01, and <0.001, respectively) (**B**) Three-dimensional response surface plots showing interactions between variables for polyphenol extraction using DES ChCl–lactic acid.

Water content critically influences the viscosity of DESs, where moderate levels enhance polyphenol extraction by optimizing hydrogen bonding capacity and mass transfer efficiency [41]. Based on previous research by Kaur et al. [42], this study investigated water content in the range of 0–50%. Under controlled conditions (molar ratio of 1:4, solid–liquid ratio of 1:20 g/mL, ultrasound duration of 30 min), TPC was evaluated across this range. As shown in Figure 4A, the maximum TPC (98.40 ± 1.30 mg GAE/g DW) was achieved at 10% water content, after which it decreased to 91.40 ± 2.03 mg GAE/g DW at 20%. A significant correlation was observed between viscosity and TPC (*p* < 0.05). Therefore, the 10% water content was selected for further optimization.

The solid–liquid ratio also plays a key role in the maximum extraction capacity of DESs under fixed conditions (molar ratio of 1:4, ultrasound duration of 30 min, water content of 20%). Based on previous research findings [42], the solid–liquid ratio range for this study was determined to be 1:10 to 1:30. As illustrated in Figure 4A, the TPC increased sharply from a ratio of 1:10 (80.56 ± 0.76 mg GAE/g DW) to 1:20 (101.22 ± 1.05 mg GAE/g DW) and then plateaued between the ratios of 1:20 and 1:30 (99.44 ± 0.54 mg GAE/g DW). An increase in the solid–liquid ratio enhances the solute–solvent concentration gradient, thereby accelerating the mass transfer of polyphenols. However, excessively high ratios can diminish ultrasonic cavitation intensity due to the increased density of solid particles [43]. To optimize extraction capacity while minimizing resource consumption, the 1:20 ratio was chosen for subsequent experiments.

Ultrasound time is another key factor affecting DES–UAE efficiency. Based on common practice in previous DES extraction studies [42,44,45], ultrasound times of 10–70 min were applied. Therefore, in this study, TPC was assessed over 30–70 min under set parameters (molar ratio of 1:4, solid–liquid ratio of 1:20 g/mL, water content of 20%). As shown in Figure 4A, TPC rose sharply from 87.46 ± 6.41 mg GAE/g DW at 30 min to 144.33 ± 2.86 mg GAE/g DW at 60 min, then decreased to 128.28 ± 6.68 mg GAE/g DW by 70 min. The initial increase suggests a high concentration gradient promoting the release of surface compounds, while the subsequent decline indicates possible equilibrium or compound degradation. Therefore, a 60 min ultrasound time was selected for further experiments.

#### 3.3.2. BBD Experimental Design

The optimal extraction process for DES–UAE was investigated using a Box–Behnken design (BBD). A four-factor, three-level BBD was established based on the preliminary single-factor tests (as shown in Table 2), which identified the optimal ranges for molar ratio (1:2), water content (10%), solid–liquid ratio (1:20), and ultrasound time (60 min). Then, a total of 29 experiments were executed, yielding the TPC results presented in Table 4. The relationship between the variables and the response was accurately described by a quadratic polynomial model (see Section 2.6).

Analysis of variance (ANOVA) confirmed the models’ high reliability for prediction. As presented in Table 4, the model was highly significant (*p* < 0.0001) with a non-significant lack of fit (*p* = 0.0913). The high R^2^ (0.9414) and adjusted R^2^ (0.8829) values further affirmed the model’s robustness in representing the relationships between the independent variables and TPC. The three-dimensional response surface plots, exemplified in Figure 4B, revealed that TPC initially increased and then decreased with increasing levels of molar ratio, liquid–solid ratio, and sonication time—a trend consistent with the single-factor experiments. Notably, significant interactive effects (*p* < 0.05) were observed for the pairs of molar ratio and sonication time (AC), molar ratio and water content (AD), and liquid–solid ratio and sonication time (BC).

These significant interactions can be interpreted through the physicochemical properties of DESs. As tunable solvents, the viscosity and polarity of DESs [46], critical for extraction efficiency, are modulated by the HBA/HBD molar ratio and water content. The significant interaction between molar ratio and water content suggests that at the optimal molar ratio of 1:2.6, a robust hydrogen-bond network forms, facilitating polyphenol dissolution. The addition of 8% water at this ratio is likely to reduce viscosity, enhancing mass transfer efficiency between the sample and solvent, and yielding superior extraction performance [47]. Simultaneously, the interaction between molar ratio and ultrasound time (68 min) indicates that ultrasound cavitation synergizes with the optimized DES structure. The cavitation bubbles disrupt cell walls, while the reduced viscosity of the optimized DESs allows more effective solvent penetration into the cellular matrix [48]. Finally, the significant interaction between the solid–liquid ratio and sonication time reflects the increasing reliance of extraction yield on ultrasound duration at higher solid-to-liquid ratios, where mass transfer limitations are more pronounced.

The optimal extraction conditions identified through response surface analysis are as follows: a molar ratio of 1:2.592, a liquid-to-solid ratio of 19.814 mL/g, an ultrasound time of 64.509 min, and a water content of 7.722%. Under these conditions, the theoretical TPC was calculated to be 195.566 mg GAE/g DW. In the validation experiments, the parameters were adjusted to a molar ratio of 1:2.6, a solid-to-liquid ratio of 1:20, an ultrasound time of 65 min, and a water content of 8%. Three parallel experiments were conducted, and the results are presented in Table 5. The total polyphenol yield from lotus leaves was measured at 187.23 ± 14.67 mg GAE/g DW, reflecting a deviation of 4.58% from the theoretical value, which indicates the model’s strong predictive capability. Furthermore, under the optimized extraction conditions, the DPPH radical scavenging capacity of the lotus leaf extract (0.92 ± 0.23 mg/mL) increased by 3.26%, while the FRAP total reducing capacity (21.56 ± 3.05 mg Trolox/g DW) exhibited a rise of 27.27%. These results indicate that the DES–UAE system established in this study achieves efficient extraction of lotus leaf polyphenols and exhibits high antioxidant activity.

### 3.4. Chemical Interactions and Morphological Characterization

**Figure 5:**
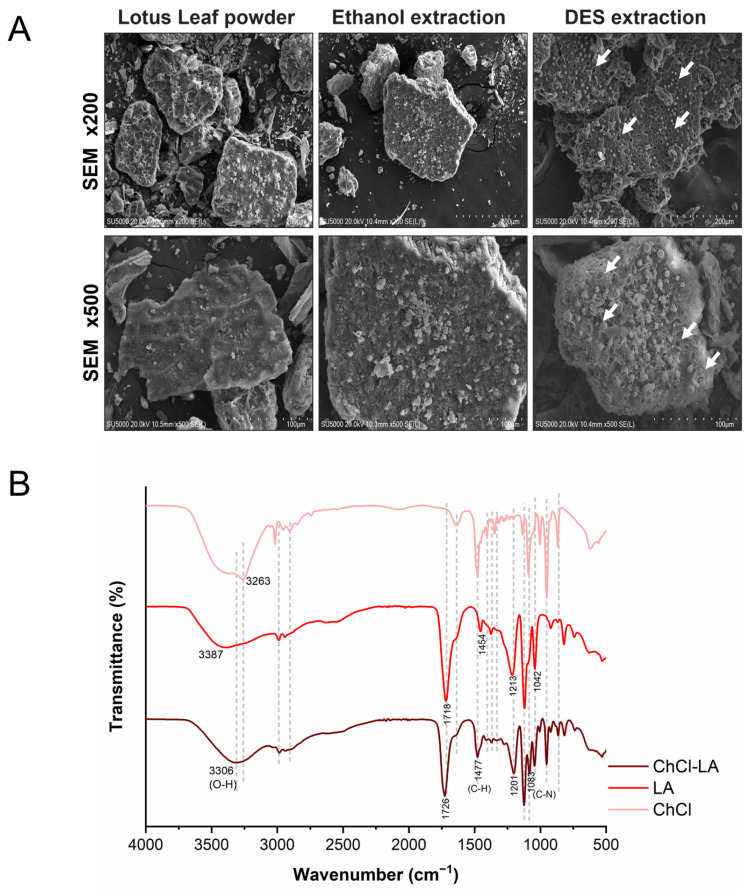
(**A**) SEM images (×200 & ×500) of untreated lotus leaf powder, and samples extracted with 75% ethanol and DES ChCl–lactic acid. Arrows indicate fragmented and porous structures. (**B**) FTIR spectra of ChCl, lactic acid, and DES ChCl–lactic acid.

This study assessed the morphological characteristics of lotus leaf powder samples that were untreated, treated with 75% ethanol, and treated with DES: ChCl–lactic acid. As illustrated in Figure 5A, the untreated powder exhibited a smooth surface, whereas the powder treated with 75% ethanol displayed a rough surface characterized by agglomerated particles. Treatment with DES: ChCl–lactic acid resulted in a markedly altered surface structure, revealing fragmented, porous structures (indicated by arrows). These distinct morphological changes are hallmark features of ultrasonic cavitation effects. Under ultrasonic influence, rapidly forming and collapsing microbubbles (cavitation bubbles) in liquids produce intense microjets and violent shear forces within confined spaces. When this energy is released near solid surfaces, such as plant cell walls, it can cause irreversible mechanical damage to the rigid cell walls [34,42]. These observations suggest that DES extraction enhances mass transfer efficiency.

FTIR spectra of the DES (ChCl–lactic acid) and its components are shown in Figure 5B. Peaks at 3387 cm^−1^ and 1718 cm^−1^ correspond to the O–H and C=O stretches of lactic acid, respectively [49]. DES formation is confirmed by C–N stretching (1020–1050 cm^−1^) and C–H bending (1454 cm^−1^) [50]. Compared to pure lactic acid, characteristic shifts occur upon DES formation: O–H stretch shift from 3387 to 3306 cm^−1^, C–H bend shift from 1454 to 1477 cm^−1^, and C–N stretch shift from 1042 to 1083 cm^−1^, respectively. These spectral shifts confirm strong hydrogen bonding between ChCl and lactic acid. These bonds disrupt the hydrogen bond networks between polyphenols and plant matrices, such as cellulose and proteins, thereby facilitating the release and solubilization of polyphenols [50]. Therefore, the DES–UAE combination utilized in this study shows considerable potential for extracting phenolic compounds from lotus leaves.

## 4. Conclusions

This study successfully employed ultrasound-assisted DES extraction technology to isolate polyphenolic compounds from lotus leaves. Following optimization, the optimal conditions established were a ChCl/lactic acid molar ratio of 1:2.6, a water content of 8%, an ultrasound duration of 68 min, and a solid-to-liquid ratio of 1:20. These conditions resulted in a yield of 187.23 ± 14.67 mg GAE/g DW, reflecting a 48.48% increase compared to the previous study. In addition, we found that the DES: ChCl/lactic acid system was significantly better than water or ethanol extraction in terms of their clearing ability of DPPH radicals (DPPH IC_50_: 0.92 ± 0.23 mg/mL) and Fe^3+^ reducing capacity (FRAP: 21.56 ± 3.05 mg Trolox/g DW).

Experiments have demonstrated that altering the HBA/HBD molar ratio and adding water changes the DES properties, which facilitates the formation of hydrogen bond networks and improves mass transfer efficiency. Additionally, extended ultrasonic treatment decreases mass transfer resistance associated with high solid–liquid ratio and disrupts cell walls through the formation and collapse of cavitation bubbles, thus further enhancing mass transfer efficiency.

This study demonstrates the potential of novel DESs as alternatives to conventional chemical solvents for the extraction of bioactive compounds. Unlike traditional chemical reagents, which are often volatile, toxic, and environmentally harmful, the novel DES: ChCl/lactic acid, are non-toxic, cost-effective, and readily accessible. These properties are consistent with the principles of sustainable development and green chemistry. Furthermore, future research will focus on the quantitative analysis of the elemental composition (e.g., Mg^2+^, Mn^2+^, Zn^2+^, Fe^2+^) of both the raw materials and the DES extracts. Investigating the potential coordination between these metal cations and the extracted polyphenols will be essential for fully elucidating the mechanisms underlying their bioactivity and stability, thereby enhancing our understanding of the DES extraction process.

Although this extraction technology has shown high efficiency at the laboratory scale, validation of the process at larger industrial scales has yet to be conducted. Consequently, future research will concentrate on kinetic studies to establish a theoretical foundation for subsequent process design and scale-up. Additionally, it will systematically compare the combined application of various physical and mechanical pretreatments with modern extraction technologies, such as supercritical solvent extraction. We believe that ultrasound-assisted DES extraction represents a novel and promising green extraction method with substantial potential for large-scale application. This approach is poised to significantly advance the high-value utilization of lotus leaves and the development of functional foods.

## Figures and Tables

**Table 1:** The common combinations for the extraction of plant polyphenols using DESs.

Plant Material	DESs Combination	Extraction Conditions	Extraction Yield	Results	Ref.
Lotus leaves	ChCl–Glycerol (1:2) ChCl–Propylene glycol (1:2) ChCl–Lactic acid (1:2) ChCl–Citric acid (1:2) ChCl–Malic acid (1:2) Bet–Glycerol (1:2) Bet–Propylene glycol (1:2) Bet–Citric acid (1:2) Bet–Malic acid (1:2) Lactic acid–Glycerol (1:2) Lactic acid–Propylene glycol (1:2) Citric acid–Glycerol (1:2) Citric acid–Propylene glycol (1:2)	50 °C/60 min (140 r/min)	Flavonoids: 126.10 mg/g Polyphenols: 126.10 mg/g	More efficient extraction yields were demonstrated by DES made of lactic acid and glycerol in a 1:2 ratio, 29% of water content, 37:1 mL/g of liquid–solid ratio, 61 min of extraction time, and 53 °C of extraction temperature.	[25]
*Capparis ovata var canescens* fruit	ChCl–Urea (1:2) ChCl–Glycerol (1:3) ChCl–Lactic acid (1:3)	30 °C/25 min, assisted by ultrasound extraction	Flavonoids: 13.04 mg QE/g DW Polyphenols: 30.55 mg GAE/g DW	The DES with a molar ratio of 1:2 between ChCl and lactic acid performed better for extraction under the ideal circumstances of 20% water, 20 mL/g, 50 °C and 30 min.	[19]
*Moringa oleifera* L. leaves	ChCl–Glycerol (1:1) ChCl–Levulinic acid (1:2) ChCl–Ethylene glycol (1:2) Bet–Levulinic acid (1:2) Bet–Glycerol (1:2) Pro–Levulinic acid (1:2) Pro–Glycerol (2:5) Pro–Lactic acid (1:1)	40 °C/30 min, assisted by ultrasound extraction	Vicenin-2: 17.6 mg/g Orientin: 23.6 mg/g	The combination of DES L-Proline and glycerol produced the highest total phenolic content. 37% water content, 144 W ultrasonic power, and 40 °C were the ideal extraction conditions.	[20]
Mulberry leaves	ChCl–Urea (1:2) ChCl–Glycerol (1:2) ChCl–Citric acid (2:1) Bet–Lactic acid (1:1) Bet–Glycerol (1:2) Pro–Glycerol (2:5) Pro–Lactic acid (1:1)	40 °C/30 min, assisted by ultrasound extraction	Polyphenols: 22.66 mg/g	The ideal circumstances for obtaining the high level of total phenolic content were ChCl: citric acid (2:1) DES with a 75% water content, 50 mg/mL of solid/liquid ratios, 40 °C and 30 min of extraction time.	[21]
*Pollen Typhae*	ChCl–Glycerol (1:4) ChCl-D–glucose (1:4) ChCl–Lactic acid (1:4) ChCl:1,2–propanediol (1:4) Pro–Glycerol (4:11)	Room temperature/35 min, assisted by ultrasound extraction	Quercetin: 0.383 μg /mg Naringenin: 0.048 μg /mg Kaempferol: 0.391 μg /mg Isorhamnetin: 3.149 μg /mg	ChCl:1,2-propanediol (1:4) produced the best polyphenols production under optimal circumstances: 30% of water content, 50 mg/mL of solid/liquid ratios.	[22]
*Lycium barbarum* L. fruits	ChCl–Glycerol (1:2) ChCl–Urea (1:2) ChCl-p-Toluenesulfonic acid (1:2)	Room temperature/1.5 h, assisted by ultrasound extraction	Myricetin: 57.2 mg/g Morin:12.7 mg/g Rutin:9.1 mg/g	The highest polyphenols production was detected in the ChCl-p-Toluenesulfonic acid (1:2) extracts over a period of 90 min and a solid/liquid ratio of 50 mg/mL.	[23]
*L. citriodora* leaves	ChCl–Lactic acid (1:2) ChCl–Ethylene Glycol (1:2) ChCl–Maltose (3:1) ChCl–Urea (1:2)	Microwaved irradiated at 65 °C for 20 min	Flavonoids: 9.02 mg/g Iridoids: 7.25 mg/g Phenylpropanoids: 17.23 mg/g	The maximum phenol extraction was obtained after 17.08 min of microwave irradiation time, 63.68 °C, 32.19% of water content with a ChCl: lactic acid (1:2) mixture.	[24]

ChCl: choline chloride; Bet: betaine; Pro: L-Proline.

**Table 2:** Parameters factor and their levels used in the study.

Parameters Factor	Symbol	Unit	Low	Medium	High
Molar ratio of HBA/HBD	A		1	2	3
Liquid–solid ratio	B		15	20	25
Ultrasonic time	C	min	50	60	70
Water content	D	%	0	10	20

**Table 3:** Composition and properties of DES combinations and their polyphenol extraction efficiency.

No.	HBA	HBD	Mole Ratio (HBA/HBD)	Water Content (%)	Heating Time (min)	Viscosity/mPa·s	Density/g/cm^3^	pH
DES-1	Choline chloride	Levulinic acid	1:2	20%	6	219.1 ± 3.56	1.13 ± 0.12	1.63 ± 0.02
DES-2		Lactic acid	1:2		3.5	167.4 ± 2.04	1.14 ± 0.13	0.72 ± 0.02
DES-3		D-glucose	2:1		11.5	618.2 ± 3.42	1.20 ± 0.02	3.88 ± 0.01
DES-4		Maltose	4:1		1	23.4 ± 1.98	1.15 ± 0.04	3.71 ± 0.02
DES-5		Glycerol	1:2		2	347.7 ± 1.23	1.19 ± 0.10	4.14 ± 0.04
DES-6		Glycerol	1:3		2	357.3 ± 5.78	1.20 ± 0.10	3.95 ± 0.03
DES-7		Urea	1:2		1.5	13.2 ± 0.68	1.16 ± 0.12	8.51 ± 0.05
DES-8	Betaine	Levulinic acid	1:2	20%	23	1055.1 ± 5.44	1.16 ± 0.15	4.88 ± 0.03
DES-9		Lactic acid	1:2		13	754.2 ± 4.32	1.18 ± 0.05	3.72 ± 0.02
DES-10		D-glucose	2:1		40	52 ± 9.78	1.27 ± 0.13	6.94 ± 0.03
DES-11		Maltose	4:1		16	95.8 ± 2.07	1.19 ± 0.14	7.52 ± 0.01
DES-12		Ethylene glycol	1:2		3	56 ± 1.22	1.12 ± 0.08	8.77 ± 0.04
DES-13		Glycerol	1:2		13	78.7 ± 1.26	1.18 ± 0.03	7.94 ± 0.02
DES-14		Glycerol	1:3		13	41.3 ± 0.74	1.20 ± 0.02	6.07 ± 0.02
DES-15		Urea	1:2		2	4.8 ± 1.66	1.13 ± 0.04	9.87 ± 0.03
DES-16	L-Proline	Maltose	4:1	20%	16	61.6 ± 1.56	1.25 ± 0.04	6.48 ± 0.05
DES-17		Glycerol	1:2		22	744.8 ± 7.87	1.29 ± 0.10	7.64 ± 0.04
DES-18		Glycerol	1:3		17	145.5 ± 4.67	1.26 ± 0.12	6.55 ± 0.02
DES-19		Urea	1:2		9	20.8 ± 5.79	1.20 ± 0.16	8.07 ± 0.01

**Table 4:** Box–Behnken design (BBD) with experimental value for lotus leaf TPC yield (mg GAE/g DW), response surface quadratic model analysis of variance (ANOVA), and response fitting statistics.

Run	BBD Experiments					ANOVA					
	A	B	C (min)	D (%)	Y (mg GAE/g DW)		Sum of Squares	Degree of Freedom	Mean Square	F-Value	*p*-Value
1	1	15	60	10	100.43	model	30,556.63	14	2182.62	16.07	<0.0001
2	3	15	60	10	177.64	A. Molar ratio of HBA/HBD	9914.46	1	9914.46	73.01	<0.0001
3	1	25	60	10	132.78	B. Liquid–solid ratio	547.56	1	547.56	4.03	0.0643
4	3	25	60	10	187.80	C. Ultrasonic time	2071.94	1	2071.94	15.26	0.0016
5	2	20	50	0	111.48	D. Water contents	2.86	1	2.86	0.0211	0.8866
6	2	20	70	0	162.37	AB	123.03	1	123.03	0.906	0.3573
7	2	20	50	20	107.31	AC	940.28	1	940.28	6.92	0.0197
8	2	20	70	20	148.10	AD	4023.09	1	4023.09	29.63	<0.0001
9	1	20	60	0	66.19	BC	1046.35	1	1046.35	7.71	0.0149
10	3	20	60	0	181.01	BD	89.7	1	89.7	0.6606	0.43
11	1	20	60	20	139.30	CD	25.47	1	25.47	0.1875	0.6716
12	3	20	60	20	127.27	A2	3342.37	1	3342.37	24.61	0.0002
13	2	15	50	10	128.79	B2	1314.7	1	1314.7	9.68	0.0077
14	2	25	50	10	166.05	C2	1502.13	1	1502.13	11.06	0.005
15	2	15	70	10	187.80	D2	9899.97	1	9899.97	72.9	<0.0001
16	2	25	70	10	160.37	Residual	1901.11	14	135.79		
17	1	20	50	10	95.16	Lack of fit	1733.97	10	173.4	4.15	0.0913
18	3	20	50	10	180.78	Pure error	167.14	4	41.78		
19	1	20	70	10	132.15	Cor total	32,457.74	28			
20	3	20	70	10	156.44						
21	2	15	60	0	112.60						
22	2	25	60	0	136.43						
23	2	15	60	20	124.54						
24	2	25	60	20	129.43						
25	2	20	60	10	190.76						
26	2	20	60	10	193.68						
27	2	20	60	10	181.56						
28	2	20	60	10	181.05						
29	2	20	60	10	179.38						

**Table 5:** Analysis of validation test results.

	Water	Ethanol	DES-2
TPC (mg GAE/g DW)	19.92 ± 3.50 ^a^	31.13 ± 9.41 ^a^	187.23 ± 14.67 ^b^
DPPH-IC_50_ (mg/mL)	7.36 ± 1.08 ^a^	4.38 ± 0.83 ^b^	0.92 ± 0.23 ^c^
FRAP (mg Trolox/g DW)	13.27 ± 0.10 ^a^	14.92 ± 1.00 ^a^	21.56 ± 3.05 ^b^

The letters in the upper-right corner of the same row indicate standard deviations, and a, b, and c indicate significant differences (*p* < 0.05).

## Data Availability

The original contributions presented in this study are included in the article. Further inquiries can be directed to the corresponding author.
